# Owner satisfaction and prognosis for return to work after pancarpal arthrodesis in working dogs in the United Kingdom: a retrospective study (2011–2020)

**DOI:** 10.1186/s13028-024-00759-5

**Published:** 2024-09-12

**Authors:** Joseph Higgins, Graham Hayes

**Affiliations:** 1 Kentdale Referrals, Moss End Business Village, A6070, Milnthorpe, LA7 7NU UK; 2 Linnaeus Veterinary Limited, Solihull, UK

**Keywords:** PCA, Pancarpal arthrodesis, Prognosis, Return to work, Working dog

## Abstract

**Background:**

Pancarpal arthrodesis (PCA) is a commonly performed procedure in the UK. However, it is not known whether working dogs in the UK and other European countries with carpal injuries that have undergone unilateral PCA are able to return to working ability as determined by the owner. Medical records from a referral hospital in the UK were reviewed retrospectively for cases of working dogs treated using unilateral PCA. Case files and radiographs were retrospectively analysed for cause of injury, diagnosis, and complications. The ability of the dog to work after surgery and owner satisfaction with the outcome were assessed using telephone questionnaire.

**Results:**

50% (5/10, 50%) owners stated their dog could perform normal duties, 4/10 (40%) could perform most duties with some allowances. Outcome was not as good for dogs working on steep, uneven fell terrain. 80% (8/10, 80%) owners rated the level of post-operative lameness as unaffected with a normal gait. 90% (9/10, 90%) owners were either very satisfied or satisfied with the outcome of the procedure, and 90% owners stated the financial investment was worthwhile.

**Conclusions:**

Unilateral PCA carries a good prognosis for working dogs with high owner satisfaction. Caution should be advised for dogs expected to work on steep, uneven fell terrain.

## Background

Workings dogs are crucial to the effective running and daily tasks on many livestock farms across Europe including Scandinavia. Musculoskeletal injuries are prevalent and a common cause of retirement of working dogs. The most common cause for early retirement was an inability to continue working due to musculoskeletal disease or injury in New Zealand police dogs and United Kingdom (UK) guide dogs [1, 2]. In the United States military working dogs, the most commonly recorded cause of death or euthanasia was degenerative joint disease [3].

A high prevalence of musculoskeletal disorders has been shown in a study of working farm dogs in New Zealand, with over 40% having at least one musculoskeletal abnormality on physical examination [4]. A later study by the same authors found almost 6/10 dogs developed at least one musculoskeletal abnormality during the course of the study, at a rate of more than four dogs per 100 dog-months at risk. Musculoskeletal diseases and injuries in dogs can lead to discomfort, pain, and a decrease in mobility. These issues can significantly impact the well-being of affected dogs and may result in a reduction in their ability to work effectively, and in turn could lead to an increased burden on other dogs on the farm, making injuries to other dogs more likely.

The carpal joint had the highest incidence rate of abnormalities in one study of working farm dogs [5], and carpal pathology was also the most common type to be recorded more than once in the same dog. The authors speculated that this type of abnormality may be more likely to persist over time than other types of abnormalities. This theory is supported by the findings that carpal injuries have been found to be common in racing Greyhounds [6, 7], while it has been suggested that carpal injuries may have been the result of overuse in sled racing dogs [8].

The canine carpus has a complex anatomy that is well documented [9], as are the mechanism of injuries and treatment options, however there is a lack of consensus regarding the optimal treatment for each injury. The canine carpus is commonly subjected to high impact forces, especially during deceleration [10] and therefore injuries are often encountered during running, jumping and falling; common activities of working farm dogs. Working farm dogs are also subjected to a number of other activities and therefore mechanisms of carpal injury such as motor/farm vehicle-related injury and being trod on by livestock, which are not seen in pet dogs.

Falling and jumping from height has been reported as the most common mechanism in previous studies of carpal hyper-extension injury [11, 12]. However, only one-third of the working farm dogs were injured by falling or jumping from a height in one study [13], with the majority of others being related to work-related activities. Similar mechanisms of injury were seen in another study of working farm dogs [14].

Injuries to the carpus include ligamentous injury, hyperextension trauma, luxation, shearing injury and articular fractures, with hyper-extension injuries reported as the most common [12, 15, 16]. However, in one population of working farm dogs [13], 5/12 dogs had obvious antebrachial luxations, while 4/12 had hyperextension injuries, therefore the cause of injury in working dogs may be differ to the pet population reflecting their additional activity levels and utilisation.

Conservative management of carpal hyperextension appears to be rarely successful due to continued and progressive hyperextension of the carpus with progressive joint instability, degenerative changes, and lameness [11, 17, 18] and treatment usually constitutes some form of arthrodesis.

Partial carpal arthrodesis (ParCA) is an option for the treatment of carpal injuries that do not involve the antebrachiocarpal joint. However, the results of ParCA for treatment of hyper-extension injuries can be disappointing, with only 50% of cases having a satisfactory result in one study [12]. Therefore, PCA has historically been recommended as the initial surgical option for any carpal injury resulting from hyper-extension [11, 12].

Numerous studies assessing the results of PCA based on working dogs in New Zealand have been performed. One retrospective study [13] found that at an average follow-up time of 18 months, 6/12 (50%) dogs could perform duties as before surgery, and 4/10 (33%) dogs could perform most former duties. Ten of the 12 (83%) owners were satisfied or very satisfied with resultant mobility and work performance of their dogs, and 11/12 (92%) owners felt the surgery was worthwhile. A subsequent prospective study [14] found that 12 months following PCA, 10/12 (83%) dogs could perform most or all duties normally. Eleven owners (92%) reported that the result of the surgery met their expectations, and nine owners (75%) were very satisfied with the outcome of the surgery. No owners were disappointed or very disappointed with the surgical outcome. The authors of both studies concluded that unilateral PCA carries a good prognosis for working dogs in New Zealand to return to work.

A follow-up study looking at longer term results of some of the dogs in one study was performed [19], and found that five of seven dogs had returned to full work, one dog had a moderate persistent lameness that prevented returning to normal work and the remaining dog did not return to normal work for reasons unrelated to the study. This follow-up study further supported the current thinking that working dogs in New Zealand have a good prognosis for return to work.

No specific investigation has been done to establish whether a similar prognosis can be expected in other working dog demographics, though in one previous retrospective study [12] assessing outcome in PCA in 40 dogs in the UK, four of these were working Border Collies. The authors found these dogs were able to go back to active work within four months of surgery.

The aim of this study was to determine owner satisfaction, long-term limb function and prognosis for ability to return to work in working farm and other dogs expected to participate in strenuous activity following PCA in the UK.

## Methods

Clinical records at one referral centre in the UK were retrospectively searched for dogs that had undergone PCA between 2011 and 2020. Inclusion criteria for the study included dogs that engaged in active work or other strenuous activity that had undergone PCA where long term follow-up was available. Exclusion criteria included any other orthopaedic or neurological condition that would have affected the dog’s ability to return to work, dogs for which incomplete data or follow-up was available.

Case records were analysed for breed, age at time of injury, bodyweight (BW), cause of injury, diagnosis, plate type and size, complications, metacarpal coverage, and any recorded complications. Immediate post-operative radiographs were assessed for implant positioning and metacarpal coverage. Follow-up radiographs were assessed for progression of arthrodesis and presence of any bone lucency indicating implant loosening. A questionnaire adapted from previous studies [13, 14] was used performed over the telephone to owners of dogs that fitted the inclusion criteria. Owners were asked to grade their dog’s working usefulness post-operative as can perform normal duties (A); Can perform most duties but allowances have to be made (B); Can perform some duties but is of limited usefulness (C); Can perform few duties, very limited usefulness (D); or not useful as a working dog (E).

### Preoperative evaluation

Radiographs were taken of both antebrachia, including from the mid-radius and ulna and the whole manus. Stressed radiographs were taken as required to obtain a diagnosis.

### Surgery and postoperative period

During the study period, two surgeons performed the arthrodesis including one ECVS and one RCVS diplomate. Dogs were premedicated with 0.03 mg/kg acepromazine (ACP; Animalcare Limited, UK), and 0.3 mg/kg methadone (Comfortan; Dechra, UK). Twenty mg/kg cefuroxime (Zinacef; GSK, UK) was administered intravenously 30 min prior to surgery, every 90 min peri-operatively and was discontinued at the end of surgery. A dorsal approach to the carpus was made as previously described [20]. Cartilage was debrided from the antebrachiocarpal, intercarpal and carpometacarpal joints and dorsal plating was used. Autogenous cancellous bone graft was harvested from the ipsilateral proximal humerus. Veterinary Instrumentation (Sheffield, UK) hybrid DCP-style PCA plates were used in all cases; dependent on the size and bodyweight (BW) of the dog. Screws were placed in the distal radius, the radial carpal bone, and the third metacarpal bone aiming for a minimum of 50% metacarpal coverage. Closure was routine as previously described [20].

Post-operative pain relief included either methadone or buprenorphine (Buprecare; Animalcare Limited, UK) as required based on subjective and objective [21] pain assessments. Non-steroidal anti-inflammatory medication was prescribed for 10–14 days following surgery, and paracetamol/codeine phosphate (Pardale-V; Dechra, UK) was prescribed for 5–7 days. All dogs were given a 5–7 days’ course of amoxicillin-clavulanate (Synulox; Zoetis, UK).

### Radiographic evaluation

Immediate post-operative radiographs were taken of the operated limb to confirm appropriate positioning of the plate and screws, ensuring bicortical engagement of all screws, the presence of a screw in the radial carpal bone and assessing for metacarpal fractures.

A modified Robert Jones dressing was applied to the limb, and the dog was recovered from anaesthesia. Dressings were changed every three days for 7–10 days unless there were any dressing-related issues. Suture removal was performed at 10–14 days post-operatively, and strict confinement was advised for six weeks.

### Follow-up

Dogs were returned to the authors’ hospital between six and eight weeks following surgery. Dogs were re-examined assessing for lameness and pain. Any post-surgical complications were recorded. Dogs were sedated and radiographs were taken of the operated carpus, assessing for evidence of bone or implant-related issues, and progression of arthrodesis was recorded.

The dogs were recommended a gradual return to normal activity, increasing on-leash activity for a further six weeks, and allowing gentle working activity approximately 4 months following surgery. Owners were subsequently contacted via telephone and the questionnaire was carried out concerning the dog’s ability to perform, and what level of, work. Assessment of the success of surgery from the owner’s perspective was recorded. Statistical analysis was not performed on the data from the questionnaire due to the low number of cases in the study.

Legal and ethical requirements with regards to the humane treatment of animals described in the study and data protection have been met according to the RCVS and the authors’ institutions’ guidelines.

## Results

### Signalment

Between 2011 and 2020, 10 dogs were identified which met the inclusion criteria. The case details are summarised in Table 1. There were nine Border Collies, with a median weight of 17.4 kg (range 13.6–23.8 kg), and one German Shepherd Dog (GSD) weighing 35 kg. There were five females and six male dogs. Mean age of all dogs at the time of surgery was six years (range 1.25-11).

Seven (7/10) dogs were used as herding and sheep trialling dogs. One (1/10) dog was used for man trailing, and two (2/10) were used for trail running.

Cause of injuries included falling off or being run over by an all-terrain vehicle (ATV) (*n* = 5), fall down a rabbit hole (*n* = 1), falling down a bank (*n* = 1), injury while running (*n* = 2), issues after previous fracture repair (*n* = 1) (Table 2). Diagnosis was made based on clinical examination and pre-operative radiographs and included carpal hyperextension injury with or without concurrent collateral ligament injury (*n* = 6), collateral ligament injury (*n* = 2), carpal luxation (*n* = 1), and dressing-related radial nerve injury after previous fracture repair at the referring vets (*n* = 1) (Table 3).

**Table 1:** Case Summaries of ten working dogs, treated using unilateral pancarpal arthrodesis (PCA) at a UK referral hospital

Case	Signalment	Time from surgery to follow-up, euthanasia or rehoming (Years)	Cause of injury	Diagnosis	Plate size	Intraop complications?	Post-operative complications	Type of work/farming	Post-operative use of dog	Q5 - How do you rate the level of post-operative lameness after healing had occurred?	Q8 - Did the result of the surgery meet your expectations?	Q9 - Overall, how would you rate your level of satisfaction?	Q10 - In retrospect, was the financial investment worthwhile?
1	2.5 year old Border Collie, 15kg	1	Unseen injury while working	Hyper-extension + collateral ligament injury	3.5/2.7	None	None	Sheep herding and trialling	Can perform normal duties	Normal gait, no lameness - YES	Yes	Very satisfied	YES
2	2.5 year-old Border Collie, 14kg	1.2	Ran over by ATV	Hyper-extension and collateraal ligament injury	3.5/2.7	None	Discharging sinus over cranial incision, cleared up with antibtiotics	Herding - Was on fell farm, needed to rehome to lowland farm as couldn’t work on fells	Can perform few duties, very limited usefulness (new owner was happy with use of the dog on lowland farm)	Intermittent non-weight-bearing	No - rehomed to a lowland farm	Disappointed	No
3	8 yeaer-old Border Collie, 23.8kg	5	Fell down a bank	Medial collateral ligament rupture	3.5/2.7	None	None	Man trailing	Can perform normal duties	Normal gait, no lameness - YES	yes	Very satisfied	Yes
4	1.25 year-old Border Collie, 17.4kg	3.3	Run over by ATV	Medial collateral ligament rupture	3.5/2.7	None	None	Herding and trialling - had to move to lowland farm	Allowances made Can perform normal duties - on lowland farm, couldn’t on fells	Normal gait, no lameness - YES	Yes	Very satisfied	Yes
5	7 year-old Border Collie, 18kg	3.6	Fell off ATV	Carpal luxation	3.5/2.7	None	None	Herding: Sheep and cattle, lowland farm	Can perform normal duties	Normal gait, no lameness - YES	Yes	Very satisfied	Yes
6	3 year-old Border Collie, 14kg	3	Fell off ATV	Hyper-extension + collateral ligament injury	3.5/2.7	Metacarpal fracture on screw tightening	None	Herding sheep dog	Can perform most duties, but allowances have to be made – especially on the fell, fine on flat fields, just need to rest her a bit	Unaffected – only see lameness after hard work	yes	Very satisfied	Yes
7	11 year-old Border Collie, 13,6kg	3	Injury while working	Hyper-extension	3.5/2.7	Metacarpal fracture on screw tightening	None	Fell running	Can perform normal duties - YES	Normal gait, no lameness - YES	Yes	Very satisfied	yes
8	10 year-old Border Collie, 21.9kg	4.6	Jumped of ATV	Hyper-extension injury	3.5/2.7	None	None	Herding - lowland	Can perform normal duties - YES	Normal gait, no lameness - YES	Yes	Very satisfied	yes
9	2.2 year-old Border Collie, 23.5kg	5	Radial nerve injury after dressing related injury after previous fracture repair	Radial nerve injury and loss of function	3.5/2.7 applied medially due to previous cranial plating on radius	None	None	Fell running	Can perform most duties, but allowances have to be made – reduced from 20–30 mile runs to 10 mile runs	Unaffected – only see lameness after hard work	Yes	Satisfied	Yes
10	4 year-old German Shepherd Dog, 35kg	2.9	Fell down rabbit hole chasing pheasant	Hyper-extension injury	4.5/3.5	None	Recurrent infections needs long courses of antibiotics	Herding - lowland sheep farming	Can perform most duties, but allowances have to be made	Constant gait abnormality, mild, intermittent lameness	Yes - as was warned infection and possible implant issues were a possible complication, this kept the outcome within expectations	Satisfied	Yes

**Table 2:** Cause of injury

Cause of injury	No.
Fall off or being run over by an all-terrain vehicle (ATV)	5
Unknown injury while working	2
Fall down a rabbit hole	1
Falling down a bank	1
Bandage related complication after fracture repair	1

The incidence of the different causes of carpal injury in ten working dogs, treated using unilateral pancarpal arthrodesis (PCA) at a UK referral hospital

**Table 3:** Nature of carpal injury

Nature of carpal injury	No.
Carpal hyperextension injury +/- concurrent collateral ligament injury	6
Collateral ligament injury	2
Carpal luxation	1
Radial nerve injury	1

The incidence of the specific carpal pathologies in ten working dogs, treated using unilateral pancarpal arthrodesis (PCA) at a UK referral hospital

### Surgery and complications

Nine (9/10) dogs were treated using a 2.7/3.5 mm VI hybrid PCA plate. Eight (8/10) of these were placed dorsally, and one (1/10) dog (with the radial nerve injury) was treated with the plate medially positioned due to an existing plate on the dorsal aspect of the radius. One (1/10) dog (the GSD) was treated with a 3.5/4.5 mm VI hybrid PCA plate.

An audible crack was noted during distal screw tightening in two (2/10) dogs. Metacarpal fracture was suspected but not visible on post-operative radiographs. No other intra-operative complications were noted, and there were no concerns on any other post-operative radiographs. All plates spanned > 50% the length of the metacarpals.

### Short-term post-operative assessment

No complications were noted in nine (9/10) dogs in the first 6–8 weeks. One dog (dog 2) developed a discharging sinus over the over the wound in the first six weeks following surgery which resolved with one course of antibiotics and did not recur.

Seven (7/10) dogs were represented for radiographic follow-up between six and eight weeks. All dogs showed progression of bony union at the arthrodesis sites with no implant-related issues. The other three dogs (3/10) were not presented for follow-up radiographs.

### Owner assessment

Average time of follow-up after surgery was 3.25 years (range 1–5 years). Seven (7/10) dogs were still alive, three (3/10) had passed away. All dogs that had passed away were due to reasons unrelated to the PCA surgery. Dog 5 was hit by a car one year after surgery, dog 7 was euthanised due to pneumonia three years after surgery, and dog 8 was euthanised due to age-related reasons four years after surgery. Dog 1 was sold to another farmer to participate in sheep trialling, which was the original plan for this dog prior to the carpal injury. Dogs 2 and 4 had been sold to other farms and were still used as working dogs.

Five out of 10 (5/10) stated their dogs could perform normal duties (A). Four out of 10 (4/10) owners stated their dogs could perform most duties, but allowances had to be made (B). One owner stated their dog could perform few duties and was of limited usefulness (D). However, when expanding on their answers, two of the owners that answered B and the owner that answered D explained their dogs struggled when working on steeper and more uneven terrain on the highland fells. However, these dogs were unaffected and able to work normally when they moved the dogs to flat lowland terrain. Two of these dogs were subsequently sold and rehomed to lowland farms. The other owner that answered B was the owner of the trail running dog, who had to reduce the length of runs from 20 to 30 miles to 10 miles.

Eight (8/10) owners rated the level of post-operative lameness after healing had occurred as unaffected with a normal gait. Two (2/8) of these owners advised there was a mild lameness only after strenuous exercise. The other six (6/8) owners noted there was no lameness or stiffness even after heavy exercise. One (1/10) owner rated their dog as having a constant gait abnormality with mild, intermittent lameness, and one (1/10) owner rated their dog as generally unaffected but with an intermittent non-weight bearing lameness.

Nine (9/10) owners stated there were no complications that were treated elsewhere. One dog (1/10 – Dog 10) developed recurrent infections requiring intermittent courses of antibiotics starting two years after surgery. This owner advised this complication was impacting the dog’s usefulness as a working dog as it was lame while active infection was present. This was ongoing at the time of writing, three years after surgery. Implant removal had not been carried out yet but had been advised to the owner and was likely to be carried out in the future.

Seven (7/10) owners stated they were very satisfied and two (2/10) were satisfied. One (1/10) owner stated they were disappointed. This was one of the herding sheep dogs (Dog 2) that had to be sold to a lowland farm, and this dog was reportedly able to work normally in its new role.

Nine (9/10) owners stated the financial investment was worthwhile, and one (1/10) owner (Dog 2) advised they thought it was not.

## Discussion

Nine out of 10 (90%) of owners reported their dog could perform all or most duties, and satisfaction was very high with 9/10 owners very satisfied or satisfied. This retrospective study provides further evidence that the majority of working dogs undergoing PCA have a good prognosis for returning to work, and that owner satisfaction after PCA surgery in this population of dogs is high. The findings are consistent with those from previous studies of working dogs in New Zealand, and confirms these findings correlate with dogs in the UK population.

The outcome of PCA in pet dogs has been described as good or excellent in previous studies [12, 22, 23]. One study reported 74% of dogs had normal limb function and 14% had slight or occasional lameness, and owners of dogs in the latter category were generally satisfied with the result [12]. Similarly in another study, 15/15 dogs had either an excellent or good outcome [23]. Owner satisfaction was high even though veterinary assessment showed mild lameness in six of 15 dogs, but 13 of 15 owners graded their dog’s gait as excellent (free of lameness). Therefore, in both these studies, owner satisfaction was high even in dogs with some lameness. This may not be the case with owners of working dogs as this could be the difference between the dogs being useful in a working capacity or not.

Four out of 10 (4/10) owners stated their dogs could perform most duties, but allowances had to be made. One (1/10) owner stated their dog could perform few duties and was of limited usefulness. When expanding on these answers, a common report was that these dogs showed lameness and struggled when being worked on the steeper and more uneven highland fell terrain. However, all owners reported these dogs could work normally when they were moved to lowland flatter terrain, either when moved within the same farm or sold to another farm.

The only owner to state they were disappointed with the procedure was one of the owners that sold their dog to a lowland farm, where the dog went on to be able to work normally. It is therefore feasible this owner would have been satisfied if the original premises had been lowland farm. This finding seems to differ from a previous study [13], where they found no significant difference for outcomes when the between different topography, and dogs did return to work on hard-hill-country farms in that country.

The mechanism of injury and nature of carpal injury in these working dogs was similar to previous reports of other working dogs [13, 14], with incidents involving ATVs making up the major of observed incidents. ATV injuries are a common cause of injury in animals and people, and one study found only 36% of users had completed formal training [24]. Improving this may reduce the incidence and of these injuries in humans and working dogs.

PCA post-operative complication rates vary from 14 to 50% [12, 13, 16, 22, 23, 25] and include implant fracture or loosening, infection, persistent pain, fracture, and wound dehiscence. It may be expected to see a higher rate of complications in working dogs compared to pet populations due to the increased demand on the arthrodesis/implants after surgery. This has not been shown in previous studies [13, 14] and was not seen in this population.

The only post-operative complication seen in this study was surgical site infection (SSI) at a rate of 20%. This is consistent with previous studies, with PCA surgical site infection rates ranging from 7.7 to 33% [16, 22, 26–28].

The infection in Dog 2 occurred at 6 weeks, was associated with patient interference, and resolved with one course of antibiotics. The infection in Dog 10 started two years after surgery. Even though radiographs were taken and there was no evidence implant failure or peri-implant lucency, this was likely associated with implant loosening and instability. Removal of the implant was recommended but had not been performed at the time of writing. Implant removal appears to be a relatively common requirement post-PCA, reported in 21% of cases in one study [25]. This owner graded the dog’s function as having a constant gait abnormality with mild, intermittent lameness, and was satisfied rather than very satisfied with the procedure because of this. It is feasible that this dog may have had superior scores if the implant had been removed prior to final follow-up, as need for implant removal was not associated with lower overall outcome and owner satisfaction in one study [14].

Prolonged external coaptation using a splint or a cast following PCA has traditionally been recommended for six-eight weeks postoperatively to reduce patient demands on the repair. A high complication rate has previously been associated with post-operative cast use [29]. 47% of the complications seen one study were cast-related [13], and the rational for external coaptation has been questioned, and has been suggested is not required [22]. Another study found that external coaptation had no protective effect on the rate of postoperative complications, but also found a high rate of external coaptation-related complications [16]. In addition to this, in the authors’ experience, farm dogs often get reduced levels of aftercare post-surgery and compliance with post-operative exercise restriction and dressing care is sometimes limited compared with pet dogs. Therefore, prolonged external coaptation is not employed at the authors’ institution and these results support this practice.

Iatrogenic intraoperative metacarpal fractures were suspected based on an audible crack during screw tightening in 2/10 (20%) cases in this study (Dog 4 and Dog 8). This is similar to previous studies [22]. These fractures were not visible on immediate post-operative radiographs. Only Dog 4 presented for follow-up at six-weeks. No problems were reported, and there were no concerns on follow-up radiographs. Dog 8 did not return for follow-up exam or radiographs. However, it is likely if this affected long term stability and therefore outcome, it would have been apparent on long term follow-up via the owner questionnaire. The authors of one study used iatrogenic metacarpal fracture as a reason to employ prolonged external coaptation [22]. The lack of further complications in dogs with suspected iatrogenic metacarpal fractures in this study may suggest this is not necessary even in these patients.

There are limitations to our study, which should be considered when interpreting these findings. These are inherent to the retrospective study design, the small patient population, possible bias from owners and lack of objective measures for assessing final outcome.

Future studies corroborating these findings alongside more objective outcome measures may add to the robustness of these findings. However, in one study looking at force-plate analysis in dogs after PCA [29], the authors found the maximal vertical loading rate of limbs following PCA was not significantly different from that of normal dogs. They concluded the gait abnormality observed is not caused by pain, but rather a mechanical gait alteration caused by the lack of ability to flex or further extend the carpus which is to be expected after PCA.

The primary intention of this study was to assess the dogs’ post-operative usefulness in a working capacity. Therefore, we deem the use of owner observations as the primary outcome assessment measure justified, as this information provides the most useful information to future clients and may aid their decision making regarding whether to perform PCA surgery or not. The finding that 90% of owners reported the financial investment to be worthwhile may help future owners in their decision-making, as in the authors’ experience economics plays a major role in these decisions.

Radiographic arthrodesis is reportedly achieved between 9 and 12 weeks for the middle carpal and carpometacarpal joints and between 17 and 30 weeks for the radiocarpal joint [30], therefore another limitation of this study is that radiographs were not taken to confirm arthrodesis. However, given the mean follow-up time of 3.25 years, the absence of reports of sudden onset of severe lameness during telephone questionnaires suggests that implant failure was unlikely, a conclusion reached in previous studies [23].

## Conclusions

The results of this retrospective study show that working dogs in the UK undergoing unilateral PCA have a good prognosis for being able to return to work. However, in contrast to previous work, the prognosis was not as favourable for dogs working on steeper, uneven highland terrain and this should be considered when advising surgery in dogs of this demographic. These results allow surgeons to provide a more accurate prognosis for working dogs requiring PCA.

## Data Availability

The datasets used and/or analysed during the current study are available from the corresponding author on reasonable request.

## Declarations

### Prior publication

Data have not been published previously.
